# Recent Advances on the Anticancer Properties of Saffron (*Crocus sativus* L.) and Its Major Constituents

**DOI:** 10.3390/molecules26010086

**Published:** 2020-12-27

**Authors:** Andromachi Lambrianidou, Fani Koutsougianni, Irida Papapostolou, Konstantinos Dimas

**Affiliations:** Department of Pharmacology, Faculty of Medicine, University of Thessaly, 41500 Larissa, Greece; mahilabrianidou@hotmail.com (A.L.); koutsfan@gmail.com (F.K.); papapostolou.iris@gmail.com (I.P.)

**Keywords:** *Crocus sativus*, saffron, cancer, anticancer activity, chemoprevention, clinical trials, patents

## Abstract

Cancer is the second leading cause of death globally with an estimated 9.6 million deaths in 2018 and a sustained rise in its incidence in both developing and developed countries. According to the WHO, about 1 in 6 deaths is due to cancer. Despite the emergence of many pioneer therapeutic options for patients with cancer, their efficacy is still time-limited and noncurative. Thus, continuous intensive screening for superior and safer drugs is still ongoing and has resulted in the detection of the anticancer properties of several phytochemicals. Among the spices, *Crocus sativus* L. (saffron) and its main constituents, crocin, crocetin, and safranal, have attracted the interest of the scientific community. Pharmacological experiments have established numerous beneficial properties for this brilliant reddish-orange dye derived from the flowers of a humble crocus family species. Studies in cultured human malignant cell lines and animal models have demonstrated the cancer prevention and antitumor activities of saffron and its main ingredients. This review provides an insight into the advances in research on the anticancer properties of saffron and its components, discussing preclinical data, clinical trials, and patents aiming to improve the pharmacological properties of saffron and its major ingredients.

## 1. Introduction

**Figure 1:**
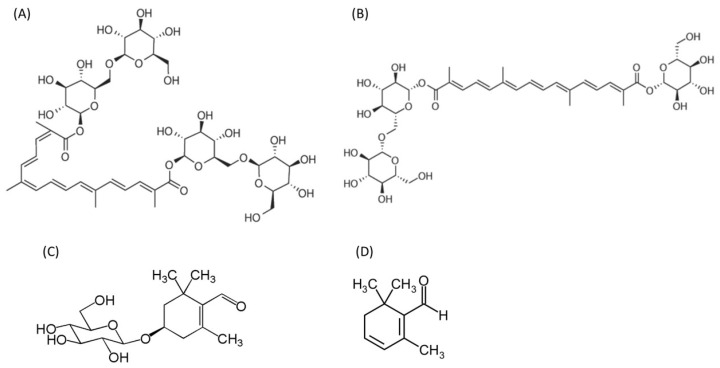
The chemical structure of the four major unique saffron ingredients. (**A**) Crocin and (**B**) crocetin are both are water-soluble carotenoid chemical compounds. (**C**) Picrocrocin is a monoterpene glycoside precursor of safranal (**D**).

Saffron, a plant product derived from the dried stigma of the *Crocus sativus* flower, is proposed to have useful biological properties [1]. Intensive research is ongoing on the importance of the health properties of saffron in its natural form [2], but a lot of interest has also been focused on the extraction, purification, and study of saffron’s major bioactive constituents, including crocin, crocetin, picrocrocin, and safranal (Figure 1). 

These phytochemicals have been reported to show beneficial effects against numerous diseases, such as diabetes [3], neurodegenerative diseases [4], cognitive problems [4], depression [5], inflammatory diseases [6], autoimmune diseases [7], digestive diseases [8], and cardiovascular inflammations [9]. The potential activity of saffron and its ingredients against cancer has been investigated as well and the results show saffron’s potent anticancer activity in preclinical settings [10], importantly without adverse effects on normal cells [11,12]. Despite the intensive research, many details on the mechanism(s) of action of saffron and its components against the progression of cancer are still unknown. It seems that saffron and its major ingredients may have a pleiotropic mechanism of action against malignant cells. There are a plethora of studies that have analyzed the effect and the action mechanism of the different ingredients of saffron extract and how their action could be optimized [13,14]; of great interest is also the fact that saffron’s major ingredients may act synergistically against malignant cells [15], suggesting that saffron extract may be more effective than its components alone. We need to note though that apart from the unique compounds that can be found only in saffron extracts, as already mentioned above, saffron possesses a plethora of other bioactive compounds, such as kaempferol and its glycosides, other flavonols such as quercetin, etc. [16].

In this review, we present and discuss from a critical point of view the most recent advances from the last five years on the anticancer properties of saffron extract and its major unique ingredients crocin, crocetin, picrocrocin, and safranal. We also discuss some critical issues that need to be urgently and properly addressed, such as harvesting and extraction conditions, storage parameters, and extract analyses, to safely and beyond any doubt come to a conclusion about the usefulness of this extract that has been a versatile medicine for the last 3500 years.

## 2. Anticancer Properties of Saffron and Its Major Ingredients

### 2.1. Effects of Saffron towards Preventing Carcinogenesis

Researchers put a great deal of effort into finding molecules that can delay carcinogenesis at the earliest possible stages of its development or even reverse cancer growth. In this context, it has been reported that saffron extract prevents tumor formation at an initial stage. In a recent study performed on hamsters that were treated with the carcinogen 7,12-dimethylbenz[a]anthracene (DMBA), oral administration of saffron at a dose of 100 mg/kg b.w./day, one week before the exposure to the carcinogen, completely prevented the formation of oral squamous cell carcinomas [17]. Similarly, the ingestion of 200 mg/kg b.w./day saffron by mice that received three topical applications of 100 nmol DMBA in 100 mL acetone delayed the onset of skin papilloma formation [18]. 

### 2.2. Saffron and Its Components as Anticancer Agents

In vitro and in vivo studies have demonstrated significant antitumor properties of saffron and its compounds. However, research is still ongoing to address questions about the particular mechanism of action of each saffron component against malignancies. The antitumor activity is mainly attributed to (i) inhibition of synthesis of DNA and RNA, (ii) inhibition or suppression of cancer cells proliferation, (iii) apoptosis, (iv) inhibition of metastasis and angiogenesis, and (v) changes in the expression pattern of oncogenes or tumor-suppressive genes (Table 1).

### 2.3. Anticancer Activity of Saffron and Its Mmajor Ingredients

#### 2.3.1. Breast Cancer

Mousavi et al. reported that saffron ethanol extract decreased MCF-7 cell viability with an IC50 of 400 ± 18.5 µg/mL after 48 h and induced apoptosis through caspase activation and Bax increment [19]. Mousavi and Baharara’s analysis showed the inhibitory effect of saffron aqueous extract on the expression of two biomarkers of angiogenesis, VEGF-A and VEGFR-2, in the MCF-7 cell line [20].

It has also been demonstrated that crocin can significantly inhibit the proliferation of MCF-7 cells, and kill cells by inducing their apoptotic cell death through mitochondrial signaling pathways activating caspase-8,9 and 3, upregulating Bax expression and conversely downregulating Bcl-2 expression, disrupting the mitochondrial membrane potential (MMP), and releasing the cytochrome c [21,22,23]. It has also been reported that crocin shows antiproliferative activity on human breast cancer cells, such as HCC70 and HCC1806, through depolymerization of spindle microtubules and production of multipolar spindles resulting in chromosomes misalignment, also inhibiting the progression of mitosis. Further studies showed that vinblastine inhibits the binding of crocin on tubulin, which indicates that crocin has the same binding site as vinblastine [24]. It is important to note that crocin did not affect normal cells that were used as controls in the experiments [24]. 

Saffron constituents, i.e., *trans*-crocin-4, crocetin, and safranal, were also found to significantly inhibit the proliferation of MCF-7 and MDA-MB231 cells [25]. Crocetin inhibited the proliferation of MDA-MB231 cancer cells similarly to *trans*-crocin-4. They inhibited the proliferation of both cell lines at concentrations higher than 200 μΜ. Safranal inhibited the proliferation of MDA-MB231 at concentrations higher than 125 μM and the proliferation of MCF-7 at concentrations higher than 500 μΜ. The MCF-7 cells that were treated with crocetin showed apoptotic DNA fragmentation in electrophoresis. Crocetin was additionally reported to inhibit invasiveness by reducing the matrix metalloproteases expression [26]. 

Nezamdoost et al. [6] tested the effect of the aqueous extract of saffron in combination with high-intensity training in female BALB/C mice bearing 4T1 cells, a mouse breast tumor model. This study was based on the idea that training in combination with some herbal components could have an anticancer function. Oral administration of saffron extract in combination with the training indeed suppressed the tumor growth resulting in a lower growth rate of the tumors in this animal group as compared to the tumors of the animals that received only the saffron extract. However, the mechanisms that mediated this delay in tumor growth remain still elusive.

Ashrafi et al. administered N-Nitroso-N-Methyluria to female Wistar albino rats, a highly carcinogenic, mutagenic, and teratogenic agent, to induce breast cancer in rats and further studied the anticancer properties of crocin [27]. They showed that crocin led to tumor growth suppression and induced apoptosis and cell cycle arrest by downregulating cyclin D1 and p21 through the p53 pathway.

Arzin and his colleagues suggested crocin as a promoting complementary antimetastatic herbal medicine for the treatment of triple-negative breast cancer. In their study, 4T1 cells were xenografted to female BALB/c mice and 200 mg/kg of crocin injected in mice thrice a week. Crocin led to tumor growth suppression with no signs of metastasis in the liver and lung of the treated animal group as opposed to the animals in the control group. Crocin was additionally found to exhibit its antimetastatic effects by regulating the Wnt/β-catenin pathway [28].

#### 2.3.2. Ovarian Cancer

Xia et al. tested the effect of crocin in the human ovarian cancer cell line HO-8910. Crocin significantly inhibited the growth rate of the cells [29]. Also, crocin raised the proportion of HO-8910 cells in the G0/G1 phase and increased the apoptosis rate. Crocin treatment was also found to increase p53 and Fas/APO-1 expression which subsequently led to the activation of the apoptotic pathway via caspase 3 activation [29].

#### 2.3.3. Gastrointestinal Cancer

Gastrointestinal cancer is amongst the most studied cancers with regard to the potential anticancer activity of saffron and its ingredients [30,31,32,33,34,35,36,37]. Studies have shown that crocin can induce apoptotic cell death in colorectal cancer cells through p53 dependent and independent mechanisms [30]. 

The anticancer effect of crocin on three human colorectal cancer cells (HCT-116, SW-480, and HT-29) has been recently reported. Crocin reduced the rate of cell proliferation but HCT-116 showed higher sensitivity to crocin than the other two cell lines. The data indicate that the sensitivity of HCT-116 to crocin is due to the wild-type p53. SW-480 and HT-29 cells have a mutant p53 tumor suppressor gene, which suggests that the anticancer activity of crocin may be linked to p53 expression [31].

Amin et al. reported data supporting the notion that crocin initiates apoptosis in HCT 116 p53 mutant cells by damaging the DNA and thus that crocin can be used for sensitizing cancer cells for other chemotherapeutic agents [32]. Crocin could lead to a G0/G1 cell cycle arrest in HCT116 wild-type cells, functioning thus as a cytostatic agent. However, in HCT116 p53 mutant cells crocin led to a G2/M cell cycle arrest and apoptosis after 48-h incubation. Also, the treatment of wild-type HCT116 cells and HCT116 p53 mutant cells with crocin and Bafilomycin A1, a lysosome and autophagosome infusion inhibitor, showed that crocin leads to programmed cell death through apoptosis, independent of autophagy [32].

Crocin was further found to be associated with reduced expression of Krüppel-like factor 5 (KLF5) and hypoxia-inducible factor-1α (HIF-1α), two important transcription factors for the development of gastric cancer, following administration in human gastric cancer cell lines AGS and HGC-27 cells [33]. Crocin also inhibited the migration, invasion, and epithelial-to-mesenchymal transition (EMT) of gastric cancer cells. Other studies support the finding that crocin can lead to an increase of the Bax/Bcl-2 ratio, activation of caspases, and also the reduction of the expression of genes such as OTC4, SOX2, NANOG, KLF4, and NUCLEOSTEMIN in AGS cells. These genes are known to regulate the cell cycle and self-regeneration in stem cells [34].

Crocetin is also reported to inhibit the proliferation of SW480 cells in a concentration-dependent manner. Crocetin induced S-phase arrest through p53-independent mechanisms accompanied by P21 induction [35].

Amerizadeh et al. injected azoxymethane in C57BL/6 mice and administered dextran sodium sulfate to induce chemical colitis— as a model associated with cancer, and to evaluate the activity of orally administered crocin, 5-FU, and their combination. Crocin indeed decreased colorectal cancer growth in an animal model but was inferior to the 5-FU. Interestingly, the arrest of tumor growth was much higher in the combination group [36].

Fujimoto et al. administered saffron extract to adenomatous polyposis coli (APC)^Min/+^ Mice (APC^Min/+^) [37]. APC^Min/+^ (C57BL/6J) mice are models for human familial adenomatous polyposis and human colon cancer. Mice were given food mixed with the saffron extract. The saffron extract (10 mg) was prepared in MeOH (10 mg/mL). In this study, it was found that saffron reduced the number of polyps in the area located next to the big intestine. This was also the area that seemed to have the highest sensitivity to the saffron extract. It is important to note that in this study the researchers administrated specific amounts of the saffron extract to the mice so that it could resemble daily intake dosage [15].

#### 2.3.4. Prostate 

In vitro experiments in prostate human carcinoma cells LnCaP, 22rv1, CWR22, PC3, and DU145 evaluated the antitumor activity of saffron aqueous extract and crocin. Both saffron extract and crocin reduced cell proliferation in all malignant cell lines tested in a time- and concentration-dependent manner, with IC50 values ranging between 0.4 and 4 mg/mL and 0.26 and 0.95 mg/mL, respectively [12].

Festuccia et al. xenografted PC3 and 22rv1 cells to investigate the antitumor effect of aqueous extract of saffron, crocin, and crocetin orally administrated [38]. Crocetin was more effective in delaying tumor growth in comparison to saffron extract and crocin. The comparative effect of treatment with extract versus crocin in terms of tumor growth did not reach statistical significance. Immunohistochemistry analyses showed that crocetin and crocin led to a reduction of epithelial–mesenchymal transdifferentiation markers, such as vimentin, N-cadherin, and β-catenin, and to an increase of cell–cell adhesion markers, such as E-cadherin, in a time-dependent manner. Additionally, crocetin, crocin, and saffron extract inhibited malignant cell invasion and migration through the downmodulation of metalloproteinases MMP-9 and MMP-2.

#### 2.3.5. Lung Cancer

Chen et al. compared the effect of crocin against the human lung cancer cell lines A549 and SPC-A1 [39]. Crocin inhibited cell proliferation and induced apoptosis in a concentration-dependent manner, accompanied by an increase of G0/G1 arrest. Crocin increased the mRNA levels of p53 and B-cell lymphoma 2-associated X protein (Bax), while it decreased B-cell lymphoma 2 (Bcl-2) mRNA expressions. Besides, crocin combined with cisplatin or pemetrexed had a stronger inhibitory effect than the single agent [39]. Thus, these results indicate that crocin could be used in combination with these chemotherapeutic agents for the treatment of lung cancer.

#### 2.3.6. Leukemia

In vitro studies have been conducted to evaluate the effect of crocetin upon the growth of various leukemia cancer cell lines such as HL60, K-562, L1210, NB4, and P388, showing that crocetin has a cytotoxic effect [40]. Moradzadeh et al. investigated the apoptogenic potential of crocetin and its underlying mechanism in acute human leukemia HL-60 cells versus normal human polymorph nuclear (PMN) cells [41]. The results showed that crocetin decreased cell viability and increased sub-G1 cell population in HL-60 cells, in a concentration-dependent manner, without significant toxicity toward normal PMN cells. Also, the expression of the caspase 3,9 and Bax/Bcl-2 ratio was significantly increased in HL-60 cells, while caspase 8 remained unchanged. It was suggested that crocetin promoted apoptosis through the induction of the intrinsic pathway. The researchers studied the effect of crocetin on these cells in comparison with ATRA (all-*trans*-retinoic acid), an anticancer chemotherapy drug, and arsenic trioxide (As_2_O_3_), which have therapeutic effect on leukemia. The toxicity of ATRA and As_2_O_3_ remains an important limitation for its use at high therapeutical doses. So crocetin may be utilized as an appropriate alternative drug against leukemia [40].

In a different study, Geromichalos et al. conducted in silico and in vitro experiments with imatinib, safranal, and crocin to study the anticancer effects of safranal and crocin in K-562 human chronic myelogenous leukemia (CML) cells [42]. Interestingly in silico studies indicated that crocin and safranal inhibit the Bcr-Abl gene expression and protein activity. Studies revealed that safranal can be attached to the Bcr-Abl protein, at the same place as the imatinib mesylate, the drug used in the treatment of CML. In vitro studies regarding the expression of the Bcr-Abl gene revealed that safranal inhibited the expression of the gene but to a lesser degree as compared to imatinib. Crocin, on the other hand, led to an increase of expression of the Bcr-Abl oncogene but also interacted with the Bcr-Abl protein and thus showed a toxic effect, through a different signal transduction pathway.

### 2.4. Use of Saffron Ingredients as Adjuvants to Chemotherapeutic Drugs

Apart from radiotherapy, chemotherapy, immunotherapy and surgery [43], scientists are also looking for alternative approaches to treat cancer and to improve the life quality of patients. Saffron’s compounds are reported to be a safe and effective treatment to reduce the toxic side effects of some conventional chemotherapeutic drugs such as tamoxifen [44], cisplatin [45], and doxorubicin [46]. Also, besides the protective features mentioned above, several studies have highlighted that the combined treatment of saffron extracts with chemotherapeutic drugs had synergistic effects, enhancing the outcome of the applied treatment. A synergistic antiproliferative and apoptotic effect of crocin and cisplatin has been reported on human osteosarcoma and lung cancer cells, for example [45]. Pretreatment with saffron significantly inhibited the induction of DNA damage (strand breaks) by antitumor drugs, like cisplatin, cyclophosphamide, and mitomycin-C, and protected against the genotoxicity of these antitumor drugs in normal cells [47].

### 2.5. The Achilles’ Heel: Bioavailability of Crocus sativus Active Compounds

The active compounds of *Crocus sativus* are considered to be quite beneficial for human health through their antidepressive, antioxidant, anticancer and antitumor effects, etc. [48]. However, all these compounds share major drawbacks, namely their lipophilic character and poor bioavailability. To start with, the effects of these bioactive constituents are related to the dose that is bioavailable and not to the dose that is ingested. Thus, bioavailability is crucial for the bioefficacy of the available drug [49]. 

In light of this, researchers put effort into improving the unfavorable features of the basic bioactive compounds of saffron, as they are characterized by their low bioavailability, stability, and absorption [50]. Better knowledge of the bioavailability of these ingredients could result in a more successful use against malignancies. There has indeed been an effort to increase the bioefficacy of the bioactive ingredients of saffron by advancing and implementing new drug delivery system methods [51,52].

Nanoparticles: this category can be divided into four subcategories due to the different kinds of carriers that have been used. There are polymeric, lipidic, inorganic, and selenium nanoparticles. At first, polymeric nanoparticles were used as encapsulating agents. The bioavailability, water solubility, stability, and targeted delivery of the encapsulated natural compounds were improved due to the small size of nanoparticles [53]. Rahaiee et al. showed enhanced crocin stability with biopolymers compared to the standard crocin [52]. Lamgroodi et al studied in MCF7 cells the effect of PLGA (poly…glycolide) upon delivering doxorubicn alone or co-delivering doxorubucin and crocetin. Co-delivey of doxorubicin and crocetin in PLGA nanoparticles resluted to higher cytotoxicity compared to other formulations or the free form of doxorubicin or crocetin [54]. Moreover, the bioavailability of crocin was demonstrated to be higher when it was encapsulated in chitosan-alginate nanoparticles [55]. 

Another form of lipid carriers, liposomes, have been used to improve the pharmacokinetic properties of saffron-derived phytochemicals. Liposomes have the benefit of being non-toxic, overcoming the poorly water-solubility limitations of the drugs and stabilizing them [56]. In 2011, Mousavi et al. showed that liposomal encapsulation of crocin enhanced the apoptogenic effects on MCF-7 and HeLa cells [57].

Another significant category of nanoparticles that have been used to improve saffron’s bioactive efficacy are inorganic nanoparticles. Inorganic nanoparticles are non-toxic, hydrophilic, biocompatible, and highly stable particles compared to organic materials. Hoshyar et al. used crocin for the synthesis of gold nanoparticles (AuNPs). Spherical, stable, and uniform AuNPs were synthesized and used to prepare crocin-AuNPs. The data demonstrated that the proliferation rate of breast cancer cells was reduced by crocin-AuNPs compared to crocin alone [58]. Interestingly, silver nanoparticles (AgNPs) are a promising saffron carrier [59].

Finally, selenium nanoparticles have been studied with regard to their capacity to carry anticancer drugs [60]. Thottumugathu et al. tried using poly(ethylene glycol)-PEG selenium nanoparticles (SeNPs) to carry crocin as a drug delivery system. In vitro studies with A549 human lung cancer cells showed that PEG-SeNPs may be promising carriers for crocin, improving its antitumor activity.

Nanostructured lipid dispersions (NLDs): nanostructured lipid dispersions retain crocin’s beneficial activity and control the release of the drug directly to the target. Specifically, NLDs can protect the crocin from degradation, control its skin diffusion, and prolong crocin’s antioxidant activity, therefore suggesting the suitability of nanostructured lipid dispersions for crocin topical administration [61].

### 2.6. Patents Based on Saffron’s (Crocus sativus) Pharmaceutical Effects in Cancer Treatment

Besides the drug delivery systems already mentioned, several patents have also been granted based on ways to improve the pharmacokinetic profiles of the saffron-derived agents. We focused on the patents that have been invented to improve saffron’s unique compounds’ action against cancer (Table 2). These patents are based on formulations that contain bioactive phytochemicals, like crocin, crocetin, and safranal, which increase their bioavailability and/or optimize their action against malignancies.

One of the main goals of these patents is the increase of the bioavailability and bioefficacy of their cargo. The inventors of patent US20130337068 report the use of carotenoids, such as lycopene, to increase the bioavailability of crocin or crocetin, and thus reduce the dose required to achieve efficacy [62]. In another patent, a purified fraction of crude crocetin, including crocetinic acid, was administered orally or intravenously for prevention, treatment, and therapy of pancreatic cancer [63]. In recent years new patents have been issued, suggesting that administration οf safranal may prevent tumor formation or restrict cancer development. In these patents, safranal was reported to be administered alone or in combination with established drugs (i.e., Sorafenib, topoisomerase-1 inhibitors, etc.) [64,65,66,67].

Crocin and crocetin have been reported to be most effective when incorporated into bipolar *trans*-carotenoids (BTCs) with a *trans*-carotenoid skeleton [68,69]. These bipolar *trans*-carotenoid salts (BTCS) are useful in improving the diffusivity of oxygen between red blood cells and body tissues. Trans isomer of sodium crocetinate indeed belongs to bipolar *trans*-carotenoid salts. It bears beneficial effects for crocetin, enhancing its solubility, and has been reported to be used to reduce hypoxia, a characteristic of malignancies [70]. 

### 2.7. Clinical Trials

As is clear from what has been presented above, a plethora of studies have been conducted upon the anticancer properties of saffron and its unique constituents, like crocin, crocetin, and safranal. Moreover, preclinical in vitro and in vivo data support the findings that the activity of these substances is targeted on malignant cells, sparing normal cells, making these natural agents ideal for developing a human therapeutic approach. However, despite this, little research has been undertaken on humans. So far only two clinical trials report the use of saffron for the treatment of cancer. There is one reported clinical study, published in the Avicenna Journal of Phytomedicine (AJP), which demonstrated the anticancer effect of saffron in combination with chemotherapy in cancer patients suffering from liver metastasis [71]. This clinical study took place in Mashhad, Iran, and it is reported to have been approved by the Ethics Committee of Mashhad University of Medical Sciences with grant number 87432. The 13 patients who participated had primary cancer, including esophagus, stomach, colon, ovarian, and breast cancers, and consumed capsules containing 50 mg of dried saffron stigma. The efficacy of this treatment was evaluated based on CT scan results. The number and size of metastatic lesions were calculated according to the guidelines of the National Cancer Institute (probably that of the USA as it is not mentioned in the publication of the trial). It is reported that 14.3% of the group showed a complete response to saffron treatment, an important outcome towards establishing the proof-of-concept for the anticancer properties of saffron. However, a larger sample size is required, as the placebo and saffron groups included only three and four patients, respectively.

Another clinical trial, referred to as the “Safety and Efficacy Study of trans sodium crocetinate (TSC) with concomitant radiation therapy and temozolomide in newly diagnosed glioblastoma (GBM)”, coordinated by INC Research, Raleigh, North Carolina, and conducted in 2013, examined the properties of trans sodium crocinate as a radio-sensitizer [72]. The trial is registered with the ClinicalTrials.gov database (http://www.clinicaltrials.gov) and its registration number is NCT01465347. A total of 59 patients with newly diagnosed GBM participated in this trial. The trial began with a Phase I run-in period to establish the safety of dosing TSC concurrently with radiation therapy and temozolomide. After a safety monitoring committee (SMC) had determined that the TSC caused no dose-limiting toxicity, Phase II was begun. In Phase II, 50 additional patients were enrolled, all receiving the established safe regimen of 0.25 mg/kg TSC, intravenously, three times a week, about 45 min before radiation therapy. Four weeks after the completion of radiation therapy, patients began chemotherapy with TMZ (150–200 mg/m^2^) for five days of the first week of a four-week cycle, continuing for six such cycles. No TSC was administered during this period of chemotherapy. During the patient visits (every eight weeks), data consisting of contrast MRI, Karnofsky Performance Status (KPS) scores, and answers to two quality of life questionnaires, were collected. Comparative tumor areas were determined based on the maximum diameters and lengths shown on the MR images. Patients were followed up for two years after their treatment began. The overall survival was analyzed using Kaplan–Meier statistics at two years, and the results were compared with the results that arose from another clinical trial in which the patients received radiotherapy plus TMZ without TSC [73]. These results strongly suggest that adding TSC during radiation therapy is beneficial for the treatment of newly diagnosed glioblastoma.

## 3. Discussion

Scientists have striven to take advantage of nature’s armamentarium and discover the beneficial properties of medicinal plants that may play an important role in human health for centuries [74]. Among such plants is *Crocus sativus* L., commonly known as saffron crocus. Studies report the beneficial action of the components of saffron against a variety of diseases and especially cancer. These findings suggest that saffron’s compounds could have potent cancer-preventing effects and antitumor activity with selective toxicity against cancer cells, without affecting the normal cells and without causing any adverse effects, such as conventional cancer treatment drugs do, or drug resistance [75].

As researchers are focused on clarifying the mechanisms through which each compound acts [76], there are plenty of data from in vitro and in vivo studies shedding light on the mechanism of their action, as has been presented above. Signaling pathways and molecules that are involved in the inhibition of cancer cell proliferation, in the triggering of programmed cell death, in the prevention of metastasis, and in the blocking of angiogenesis are the major subjects of all the research [77]. Despite the promising results that have emerged from these extremely important and sound studies, we have to underline that there is a lack of human clinical trials. We cannot draw any definite conclusions so far, as the information about the beneficial effects of saffron’s unique ingredients against malignancies is still at the preclinical level. A study conducted in Iran by Hosseini and his colleagues [71] reported encouraging results in patients with liver metastases, but without examining the course of primary cancer after saffron administration, and the authors emphasized the small sample size of their study. The second and maybe most important clinical trial, as we mentioned above, was carried out based on the crocinate patents, with a sufficient number of participants, and reported encouraging results for administration of trans sodium crocetinate during radiation therapy in patients with glioblastoma [72]. What is certain is that more human clinical studies with a sufficient number of participants are required to confirm both the actions of saffron and its main ingredients and the safest dose of saffron administration with the best outcome against cancer.

Besides the need for clinical trials, another urgent need is the determination of specific protocols and approved guidelines regarding some practical but extremely important issues that are required to ensure consistency and repeatability of the results of all conducted studies. 

Since 2011, guidelines for the analysis of saffron’s major bioactive compounds have been established by the International Standards Organization (ISO 3632). According to ISO 3632, crocin, picrocrocin and safranal are responsible for the color, the flavor, and the aroma of saffron, respectively. This ISO defines specific procedures to determine the concentration of these compounds by spectrophotometric analyses and the variations in concentrations of substances by which saffron quality can be classified have been established. These values are defined as a direct reading of the absorbance of a 1% aqueous solution of dried saffron at 257, 330, and 440 nm using a 1 cm pathway quartz cell [78]. From the analysis of the literature we undertook, it seems that so far the majority of researchers have not followed the ISO guidelines. Thus, a strong recommendation to classify all the saffron samples to be studied, according to ISO trade specifications and quality parameters [79], so that the results obtained are comparable, should be made. By adapting and applying these techniques as established by ISO, wrong labeling and fraud with low-quality saffron material could be militated and limited. Besides, if the isolation and characterization of the main components of saffron are carried out according to the ISO specifications, it will be much easier to analyze bioavailability and the bioefficacy because it will be more consistent with their pharmacokinetic properties. 

Analyses of the accurate determination of saffron’s composition are however only a piece of a bigger puzzle. *Crocus sativus* L. is a 20–30 cm tall flower blooming in autumn. Generally, saffron is adaptable to temperature and can grow on soils varying from sandy to well-drained clay loams. It blooms in autumn and spends a long period of dormancy in the summer. The flowering period is usually between 15 October and 20 November and may vary depending on the temperature. This period is also the harvest period of saffron. Saffron produces stigmas annually and these parts of the plant are the ones used for medical purposes. The Mediterranean environment is considered worldwide as the best region to produce saffron with regard to its quality, which is attributed to many factors [78,80]. The little available information on flowering phenology in saffron has related it to environmental conditions like temperature radiation, water availability, or nutrients [81]. Temperature and soil water content trigger flowering whereas unitary stigma weights negatively correlate with the flower number per area unit. Higher air temperature and no excessive rain during the flowering period generate the best high-quality stigma yield [82]. Saffron yield is a parameter that also depends on agronomic aspects [83]. For example, soil preparation before planting is necessary. The field should be plowed four to five times to a depth of 30–35 cm to bring the soil into fine tilth. Planting time with the appropriate crop density is important for better yield performance. In Greece, for example, corms are planted in furrows at a distance of 25 × 12 cm, whereas, in Italy, where saffron is planted annually, the best yields of flower and corm productions were obtained by planting corms in furrows at a spacing of 2–3 cm. Recommended planting depths for corms vary from 7.5–10 to 15–22 cm. Finally, harvesting the flowers and separating the stigmas from the flower is a difficult operation. The flowers should be picked exactly when they are fully bloomed. The harvesting must begin shortly after dawn because upon exposure to the sun stigmas lose color and flavor. A two-year study recently published evaluated the effect of soil texture and chemical properties (pH, electrical conductivity, organic carbon, organic matter, total, and active lime) on saffron growth, yield, and quality [82]. The soil conditions were found to be essential for the high quality characteristics of the spice. The best performance, in terms of stigma, observed in soils characterized by sandy loam or loam texture, respectively with neutral-sub alkaline pH and a good amount of organic matter. Similar results were also observed by Khorramdel et al., who reported that stigma yield in sandy loam soil was 49% higher than in greenhouse experiments [84]. It is thus obvious that pedoclimatic factors may impact the quality of the final product i.e., saffron extract, which will further affect the outcome of any bioefficacy studies that follow. 

Postharvest treatment is necessary to convert stigmas into saffron spice. Three molecules are the major determinants of the properties of saffron i.e., crocin, picrocrocin and safranal, responsible for the color, the flavor, and the aroma of saffron, respectively [85]. However, the concentrations of these saffron ingredients (and therefore the aroma, the color, and the flavor of saffron) depend on the drying and storage conditions, which are decisive for the quality of the spice [86,87]. For example, saffron stigmas that have been dried in an oven will not have the same properties as others that have been dried in the sun or an airy place in the shade [88]. The highest coloring strength is obtained when saffron is treated at higher temperatures and lower times. Also, a higher amount of safranal (aroma) and crocin (color) is obtained at high temperatures. Picrocrocin concentration was not found to be affected at different temperatures in drying methods [86]. Tong et al. concluded that drying treatment at lower microwave power and over a longer time benefitted the quality of saffron. In this work, the authors suggested that the highest quality of saffron is obtained when fresh saffron is dried at a high temperature, no more than 70 °C, using an electric or vacuum oven [89].

Saffron is well known to be very hygroscopic, oxidizing, darkening, and losing its aroma when exposed to moisture, so storage conditions are essential to preserve the quality of the product if it needs to be stored and not used immediately. The studies by Tsimidou and Biliaderis [90] and Bolandi and Ghoddusi [91] reported high humidity results in the degradation of crocin and picrocrocin. Sereshti et al. concluded that the storage time/duration affects the saffron quality [92]. In this study, the relative concentration of the saffron metabolites in freshly dried and two-year-stored saffron samples prepared with ISO 3632 were evaluated. Freshly dried samples had higher levels of crocin and picrocrocin, while the stored samples were more abundant in safranal as the main saffron aroma agent, reflecting a negative correlation between them. 

The biocomponent quality of saffron, also depends on the extraction methods: duration, solvents, and extraction temperature may significantly affect the composition and the quality of the extract [93]. In this study, it was reported that a long-lasting extraction, e.g., 24 h, caused the loss of coloring strength. With alcoholic extracts, a better coloring strength was obtained as compared to aqueous extraction. The highest coloring strength values were obtained with extracts prepared with 50% water: ethanol solvent. A water/ethanol solvent was found to be better than water/methanol, and this last to be better than water alone because the polar carotenoids of saffron are not easily soluble in cold water, while they are soluble in alcoholic solutions. Notably, the concentrations of the molecules in the solutions were determined according to the ISO 3632 standards. In agreement with these findings, Gazerani and his colleagues [94] showed that the optimal parameters to extract the compounds from saffron were an extraction solvent of 50% aqueous ethanol and extraction condition of 5 h at 25 °C. The authors subsequently determined the crocin, picrocrocin, and safranal contents of the extract, following the ISO 3632 guidelines. Under these conditions, the absorbances for crocin, picrocrocin, and safranal were 423.9, 49.51, and 133.1, respectively, as compared to 125.4, 24.99, and 67.18, respectively, for distilled water, which was the control, suggesting a much higher concentration in the 50% aqueous ethanol extract [94]. Generally, conventional extraction methods, which include Soxhlet extraction, vapor or hydrodistillation and maceration or solvent extraction, use huge amounts of organic hazardous solvents, are not selective, have long extraction times, and in some cases extirpate thermolabile/heat sensitive compounds. To overcome these drawbacks, novel extraction methods have been developed, known as “green methods”. Enzyme-assisted extraction, ultrasound-assisted extraction or sonication, microwave-assisted extraction, and emulsion liquid membrane extraction are examples of green extraction techniques, which exhibit appropriate potentials to extract saffron bioactive compounds [95]. For example, it is reported that under optimized emulsion liquid membrane extraction, more than 90% of saffron bioactives (i.e., safranal, picrocrocin and crocins) were collected into the aqueous phase, thus underlining the importance of a proper extraction method.

Low absorption of saffron’s active compounds is another significant obstacle and thus the method of their administration may greatly affect its bioavailability and biodistribution [96]. To address the low bioavailability, new delivery methods with the aid of advanced drug delivery systems are being developed, so that saffron’s bioactives can be delivered with increased efficacy. In this context, many patents focused on methods to improve the efficacy of saffron’s ingredients against cancer have been issued. 

In conclusion, we herein reviewed advances over the last five years with regard to the anticancer properties of saffron and its major ingredients, crocin, crocetin, picrocrocin, and safranal, recent improvements addressing their poor pharmacokinetic properties, patents, and clinical trials geared towards evaluating their use as potential agents to fight cancer. Major improvements have been achieved with the aid of pharmaceutical technology and the use of novel drug delivery systems. In general, all the results that emerge continue to be very encouraging, especially these from the two clinical trials. In our opinion, this is precisely the next major step that must be emphasized and toward which more effort from the scientific community should be directed: more clinical trials should be set up to get the final proof-of-concept for the potential of saffron and its ingredients as anticancer agents. However, these clinical trials should be conducted only after adopting strict and very specialized protocols for the preparation, storage, and use of saffron and its ingredients to ensure the use of only high-quality saffron material and its consistency.

## Figures and Tables

**Table 1:** Major studies on the anticancer effects of saffron extract and its major ingredients in cell lines and animal models of cancer.

Bioactive Compound	Cell Line/Animal Model	Suggested Mechanism of Action/Outcome	References
Saffron ethanol extract	Breast cancer cells MCF-7	Caspase activation, upregulation of Bax expression. Apoptosis.	Mousavi et al., 2009
		Downregulation of VEGFR expression. Cell proliferation and angiogenesis inhibition	Mousavi and Baharara, 2014
Safranal, crocin	Myelogenous leukemia cells K-562	Downregulation of Bcr-Abl expression	Geromichalos et al., 2014
Safranal, crocetin, crocin	Breast cancer cells MCF-7, MDA-MB 231	Cell proliferation inhibition	Chryssanthi et al., 2007
Crocetin	Breast cancer cells MCF-7, MDA-MB 231	Downregulation of metalloproteases expression. Cell invasion inhibition, apoptosis	Paper et al., 2000
Crocetin	Colon cancer cells SW480	Arrest of cells in the S phase, upregulation of P21 expression. Cell proliferation inhibition	Li et al., 2012
Crocetin	Leukemia cells HL60, K-562, L1210, NB4, P388	Activation of the intrinsic apoptotic pathway	Moradzadeh et al., 2019
Crocetin, crocin	Lung adenocarcinoma cells A549, SPC-A1	Upregulation of the p53 and Bax mRNA levels; downregulation of the f Bcl-2 mRNA levels. Apoptosis	Chen et al., 2015
Crocin	Breast cancer cells MCF-7	Caspase-8,9 and 3 activation, Bax/Bcl-2 ratio increase, mitochondrial membrane potential disruption, cytochrome c release. Cell proliferation inhibition, apoptosis	Lu et al., 2015
			Bakshi et al., 2016
			Mostafavinia et al., 2016
Crocin	Breast cancer cells HCC70, HCC1806	Microtubules depolymerization. Cell proliferation inhibition	Hire et al., 2017
Crocin	Ovarian cancer HO-8910 cells	Arrest of cells in the G0/G1, upregulation of p53, Fas/APO-1, and Caspase-3 expression. Apoptosis	Xia et al., 2015
Crocin	Gastric cancer cells AGS	p53 dependent and independent mechanisms. Apoptosis	Hoshyar et al., 2017
Crocin	Colorectal cancer cells HCT-116, SW-480, HT-29	p53 dependent action. Cell proliferation inhibition	Aung et al., 2007
Crocin	Colorectal cancer HCT116	p53 dependent action, arrest of cells in the G0/G1, down-regulation of Beclin 1, and Atg7 expression. Apoptosis	Amin et al., 2015
Crocin	Gastric cancer cells AGS, HGC-27	Downregulation of Krüppel-like factor 5 (KLF5) and hypoxia-inducible factor-1α (HIF-1α) expression. Cell proliferation inhibition	Zhou et al., 2019
Crocin	Gastric cancer cells AGS	Downregulation of OCT4, KLF, SOX2, NANOG, and Nucleostemin expression. Apoptosis	Akbarpoor et al., 2020
Crocin	Prostate cancer cells BPH-1, LnCaP, 22rv1, CWR22, PC3, and DU145, LAPC-4, C4–2B	Arrest of cells at the G0/G1, activation of the intrinsic apoptotic pathway	D’Alessandro et al., 2013
Saffron aqueous extract	4T1 cells were xenografted in mice	Changes in p53 expression. Inhibition of tumor progression	Nezamdoost et al., 2020
Crocin	Administration of N-Nitroso-N-Methyluria to female Wistar albino rats	Downregulation of cyclin D1 and p21 expression. Inhibition of tumor progression, apoptosis.	Ashrafi et al., 2015
Crocin	4T1 breast cancer cells were xenografted in mice	Controlling metastasis via Wnt/β-catenin pathway. Inhibition of tumor progression, antimetastatic effect	Arzi et al., 2018
Crocin	T24 cells xenografted in BALB/c nude mice	Downregulation of Survivin, Cyclin D1 and upregulation of the Bax/Bcl-2 ratio. Apoptosis	Zhao et al., 2008
Crocin	Azoxymethane and dextran sodium sulfate to induce chemical colitis associated with colorectal cancer in mice	Inhibition of tumor progression	Amerizadeh et al., 2018
Crocin	Adenomatous polyposis. ApcMin/+ mice: models for human familial adenomatous polyposis	Decrease in the number of intestinal polyps	Fujimoto et al., 2019
Crocin, crocetin	Prostate cancer cells PC3 and 22rv1 xenografted in male nude mice	Downregulation of N-cadherin and b-catenin expression, upregulation of E-cadherin expression. Inhibition of tumor progression, cell invasion and migration	Festuccia et al., 2014

**Table 2:** Patents based on saffron and its major ingredients and their intended applications.

Patent	Title of Patent	Saffron Component	Subject of the Patent/Application	Inventors (Year Patent Issued)
AU2019264659	Combination therapy for cancer (i.e., a TOP inhibitor)	Safranal	Potent method of treating, suppressing, or reducing the severity of a liver cancer	Amin, A. (2019)
AU2019264660	Method of liver cancer treatment with safranal-based formulations	Safranal	Potent method of treating, suppressing, or reducing the severity of a liver cancer	Ala’a Al Hrout, Amin, A. (2019)
US10568873	Safranal–Sorafenib combination therapy for liver cancer	Safranal	Potent effective treatment for liver cancer	Amin, A., Al Mansoori, A., Baig, B. (2020)
US20200276133	Prevention of liver cancer with safranal-based formulations	Safranal	Potent method of preventing the formation of liver cancer in a subject	Amin, A. (2020)
US20130337068	Carotenoid particles and uses thereof	Crocetin or crocin	Increase of the bioavailability of the delivery molecules	Petyaev, I. (2013)
US9889105	In vivo method for treating, inhibiting, and/or prophylaxis of cancer, such as pancreatic cancer	Crocetin or crocin	Possible inhibition of tumorigenesis in vivo	Dhar, A., Gutheil, G.W. (2018)
US20040116729	Bipolar *trans*-carotenoid salts and their uses	Crocetin or crocin	Improvement of crocin’s and crocetin’s efficacies	Gainer, J., Grabiak, R. (2013)
US6060511	*Trans*-sodium crocetinate (TSC), methods of making and methods of use thereof	Crocetin	Enhancement of crocin’s solubility and increase of bioavailability and radio-sensitizer in in vivo studies	Gainer, J.L. (2000)
US2015352068	Oral formulations of bipolar *trans*-carotenoids (BTCs)	Crocetin	Oral dosage forms of BTCs in chemotherapy	Gainer, J.L., Murray, R. (2015)

## Data Availability

Not applicable.
